# From Pond to Plate: Tailored Functional Proteins for Next-Gen Microalgal Food

**DOI:** 10.3390/foods15142428

**Published:** 2026-07-08

**Authors:** Johannes Zoehrer, Xuze Wang, Adelheid Stopfer, Peren Tarlabasi, Cecilia Castells, Carmen Cuéllar Fernández, Paula Rodríguez de Juan, Daniel Garbe, Thomas Brueck, Dania Awad

**Affiliations:** 1 Werner Siemens-Chair of Synthetic Biotechnology, TUM School of Natural Sciences, Technical University of Munich (TUM), Lichtenbergstraße 4, 85748 Garching bei München, Germany; go29qep@mytum.de (J.Z.); xuze.wang@tum.de (X.W.); heidi.stopfer@gmx.de (A.S.); ge64hom@mytum.de (P.T.); go29naz@mytum.de (C.C.); carmen.cuellar@tum.de (C.C.F.); go72kuq@mytum.de (P.R.d.J.); daniel.garbe@tum.de (D.G.); 2 TUM AlgaeTech Center, Department of Aerospace and Geodesy, School of Engineering and Design, Technical University of Munich (TUM), Willy-Messerschmitt-Str. 1, 82024 Taufkirchen, Germany

**Keywords:** microalgae, microalgae cultivation, protein harvesting, food, food security, population

## Abstract

Climate change, increasing disease pressures, and rising global protein demand challenge future food security and highlight the need for sustainable alternative protein sources. Microalgae have emerged as a promising complementary feedstock due to their rapid growth, high protein content, favorable amino acid profiles, functional properties, and low land requirements. However, despite extensive research progress, industrial scale-up and economic feasibility of microalgal protein production remain limited. This review critically assesses the current state of microalgal protein research across the production chain, including strain selection, cultivation, harvesting, protein extraction, processing, and food applications. While plant-derived protein isolates are already widely used to improve food nutritional and functional properties, microalgal protein isolates remain underexplored in real food matrices, with most applications relying on whole biomass incorporation. Downstream processing represents a major economic bottleneck, particularly for high-purity protein products. The review identifies key knowledge gaps, evaluates socio-economic potential, and highlights limitations in current sustainability assessments. Although microalgae are unlikely to serve as a stand-alone solution to global protein demand, they offer strong potential as a complementary, resource-efficient protein source. Realizing this potential will require interdisciplinary research, improved technoeconomic analyses, supportive regulatory frameworks, and targeted investment to enable industrial implementation and contribute to a more resilient and climate-friendly food system.

## 1. Introduction

The rapid growth in human population, coupled with the depletion of fossil-based resources, has created dual challenges: addressing human-induced climate change driven by greenhouse gas emissions (GHG emissions) while ensuring global food security. Addressing these interconnected challenges necessitates a transformative shift that both reduces carbon emissions and maintains political and economic stability. This is particularly essential in countries of the Global South and can be achieved by coordinated global action. To that end, innovations across diverse sectors require significant scientific efforts to fundamentally reconsider the origin and use of our resources, aimed towards the adoption of a circular economy model [1,2].

Food production, particularly livestock farming, is a major contributor to global greenhouse gas emissions, accounting for a significant share of total emissions [3]. Animal-based protein production is generally less land- and resource-efficient than plant-based alternatives, resulting in higher emissions per unit of food and increased pressure on land use [4,5]. In contrast, plant-based and alternative protein systems can provide higher protein yields per area, thereby reducing the need for agricultural expansion [6]. Consequently, there is growing interest in sustainable protein sources, including microbial, plant-, insect-, and fungal-based systems as complementary solutions [7,8].

Algae are a diverse group of photosynthetic organisms that play a crucial role in maintaining Earth’s carbon cycle and oxygen production [9,10]. In parallel, they play a pivotal role in the sequestration of carbon dioxide by converting CO_2_ into organic carbon, thus influencing the global carbon cycle and climate regulation. They are classified as macroalgae (seaweeds) and microalgae [9,11]. Macroalgae are multicellular macroscopic organisms exhibiting a wide range of sizes, with some species reaching more than 50 m in length [9,12]. Representative edible macroalgae include the green algae Ulva [13] and Codium [14], the red algae Porphyra (nori) [15] and Palmaria [16], and the brown algae such as Saccharina and Laminaria [17], many of which have a long history of human consumption, particularly in Asia. Microalgae, by contrast, are unicellular organisms that dominate aquatic primary production [18]. In addition to their ecological roles, including sustaining aquatic food webs and forming symbiotic relationships with other organisms, such as corals and sponges [19], as well as fungi [20], microalgae are distinguished by their exceptional ability to assimilate inorganic carbon and other nutrients from their environments (e.g., nitrogen and phosphorus). Through photosynthesis, they generate protein-rich biomass that is subsequently transferred through the food chain as they are consumed by zooplankton and subsequent higher organisms [10,21,22]. In addition to their presence in freshwater and marine ecosystems [9], microalgae can also inhabit extreme environments, such as hot springs and hypersaline lakes or polar regions [23,24]. This ecological adaptability makes them particularly valuable for biotechnological applications. Given their inherent biological potential, they are regarded as promising platforms in biotechnology that can complement existing agricultural and industrial systems, with the potential to revolutionize various application fields ranging from sustainable energy production and the production of valuable biochemical substances to wastewater treatment and food production [25].

Microalgae are increasingly recognized in scientific discourse as emerging alternatives/complementary sources alongside established protein sources such as legumes (soy and peas) and insects. They offer a rich source of proteins providing essential amino acids, along with vitamins, minerals, and bioactive compounds [26]. However, despite a wide array of algal food products and extensive research literature (algal cultivation, protein composition, and technofunctionalities, etc.), utilizing microalgal protein isolates/concentrates as raw material for different food applications remains poorly documented and underexplored commercially. Most recent reviews focus either on biomass-based products or nutritional composition [27,28,29,30,31]. Microalgal protein isolate production remains a complex and multidisciplinary challenge that requires further technological and scientific advances across the entire production chain. For many food applications, highly purified protein fractions are preferred to minimize undesirable effects associated with whole-cell biomass incorporation and to ensure consistent techno-functional performance. However, protein isolation and purification substantially increase process complexity and costs, while biomass production itself remains one of the major economic bottlenecks. Despite these challenges, continued research efforts and targeted funding initiatives are essential to accelerate technological development and improve the economic feasibility of microalgal proteins. This review provides a holistic overview of current microalgal protein production and processing strategies, identifies key knowledge gaps, and highlights priority areas for future research. Furthermore, it emphasizes the need for coordinated policy support, investment in scale-up technologies, and the development of standardized processing and safety frameworks to facilitate the transition of microalgal proteins from niche applications to commercially viable and sustainable food ingredients.

## 2. Microalgal Diversity and Current Food-Grade Strains

### 2.1. Taxonomic and Physiological Diversity of Microalgae for Protein Production

Microalgae, the focus of this review, exhibit a remarkable taxonomic diversity. Prominent representatives include green algae (Chlorophyta) such as *Chlorella*, *Chlamydomonas*, and *Scenedesmus* [32], red microalgae such as *Porphyridium* and *Galdieria* [33], diatoms such as *Phaeodactylum*, *Cyclotella*, and *Thalassiosira* [34], and cyanobacteria such as *Synechocystis*, *Arthrospira*, *Nostoc*, and *Synechococcus* [35]. Although cyanobacteria are prokaryotic and not algae in a strict taxonomic sense, they are commonly grouped with microalgae in applied and biotechnological contexts due to their photosynthetic metabolism and similar cultivation and utilization strategies [36]. While approximately 40–50,000 microalgae species have been formally described, estimates suggest that up to 800,000 species may exist [9].

**Figure 1:**
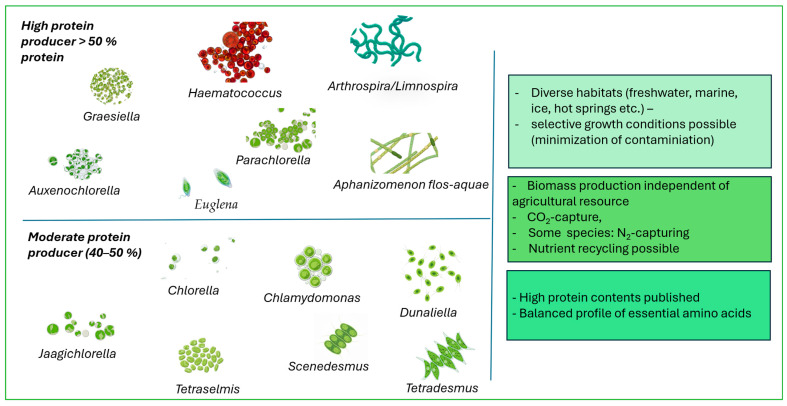
High protein producers mentioned in the EU Novel food catalog.

Microalgae are characterized by high biomass productivity and protein contents ranging from 27 to 70% of dry weight and provide a well-balanced profile of essential amino acids, making them a promising vegan protein source [26]. Currently, only a select number of microalgal species have achieved regulatory approval for human consumption, including *Arthrospira platensis* with a reported protein content of 53–70% [26], *Chlorella vulgaris* (51–58%) [37], *Dunaliella salina* (rich in beta-carotene and a protein content of 19–41%) [38,39], and *Haematococcus lacustris* (astaxanthin producer and a protein content of 25–60%). However, despite the vast diversity of putative microalgal strains, only a small proportion of species have been studied for their potential in biotechnology and protein production [9,11,40,41]. This highlights the significant untapped potential for future discoveries of valuable strains and the importance of the research in strain discovery and characterization. In-depth research on currently studied strains is essential to unlock the broader application potential of those yet to be explored—a process that can be accelerated when suitable cultivation systems and downstream processes are already in place. Identifying strains that can be efficiently cultivated under diverse environmental conditions could contribute to more sustainable protein production systems, for example, by reducing freshwater demand through the use of marine strains, decreasing fertilizer requirements, or lowering the energy input required for cell disruption through naturally weaker cell walls. For instance, food-grade *Aphanizomenon* species with a protein content of 60–70% are able to fix atmospheric nitrogen in addition to CO_2_ [42]. The discovery of new strains with superior protein content and quality could support the food industry, address global nutritional challenges, and promote more sustainable food production practices. This potential can be further enhanced through random (mutagenesis) and targeted genetic strain optimization [43,44]. Figure 1 shows microalgae with moderate and high protein content.

### 2.2. Regulatory Status of Food-Grade Microalgae

Food legislation plays a central role in determining which microalgal species can be used for human consumption, as it governs safety, nutritional evaluation, and market authorization. Regulatory frameworks differ globally: in the United States, approval is managed by the Food and Drug Administration (FDA); in China by the National Health Commission (NHC); in Australia by Food Standards Australia New Zealand (FSANZ); and in the European Union (EU) under the Novel Food Regulation (European Union) 2015/2283. Foods consumed before 15 May 1997 may face simpler approval in the EU and are declared as non-novel foods [45]. Within the EU, the European Food Safety Authority (EFSA) plays a key role in the scientific risk assessment of novel microalgal food products by evaluating their compositional data, production processes, toxicological safety, allergenic potential, and anticipated intake levels. Mendes et al. provide a concise overview of algae food regulations, with a main focus on the EU [45]. According to Su et al., only a limited number of microalgal species have obtained Generally Recognized as Safe (GRAS) status from the U.S FDA, including *Chlorella* sp., *Crypthecodinium cohnii*, *Dunaliella* sp., *Haematococcus* sp., *Porphyridium cruentum*, *Schizochytrium* sp., and *Arthrospira* sp. We acknowledge that *Schizochytrium* sp. is a thraustochytrid (heterotrophic protist) and not a true microalga [46]. However, it is noteworthy here as its oil is commercially marketed and widely used as ‘microalgae oil’ in food applications. This limited number of registrations is a direct consequence of the time- and cost-intensive comprehensive scientific evaluation required for GRAS determination [47]. Table 1 lists EU-approved species defined as ‘non-novel food’, and Table 2 lists species declared as ‘novel food’. The tables present their protein contents and provide links to available literature on amino acid profiles. A comprehensive review of all listed microalgal species revealed inconsistencies and gaps in the current taxonomic nomenclature, consistent with the published Novel Food Status catalog. Interestingly, despite the growing number of approved microalgae and the current research focus on microalgal proteins, data on their protein content and the amino acid profile of several mentioned subspecies remain fragmented and inconsistently quantified across different methods, emphasizing a significant need for standardization in algal research.

## 3. Predominance and Challenges of Whole-Biomass Applications in Food Systems

In the context of food applications, microalgal products can be categorized into whole biomass, protein concentrates, and protein isolates. Whole biomass refers to minimally processed dried or paste forms containing the full cellular matrix. Protein concentrates typically contain approximately 50–80% protein (dry weight) and retain substantial non-protein components such as carbohydrates, lipids, and pigments, whereas protein isolates are highly purified fractions, often exceeding 85–90% protein, with most non-protein constituents removed. Most studies on microalgae in food applications have focused on the direct incorporation of dried microalgal biomass, typically as powders or flours, rather than on protein concentrates or isolates. This approach is generally restricted to low inclusion levels, as pigments and flavor-active compounds can markedly affect food appearance and taste, limiting broader application. Table 3 provides an overview of the diverse applications of microalgae in food systems, highlighting key themes such as functional benefits, technological feasibility, and sensory challenges.

Across all applications, dosage dependency appears to be a critical factor. Functional benefits tend to peak at low to moderate concentrations (1–3%), beyond which negative sensory impacts and structural changes in the food matrix become more prominent. Furthermore, untreated biomass remains enclosed by rigid cell walls, leading to significantly reduced digestibility, which is strongly strain-dependent [107]. This limits the effective nutritional integration of whole-cell algal biomass in food applications.

Aldehydes such as (E,E)-2,4-heptadienal, as well as volatile sulfur compounds such as dimethyl sulfide, are typical undesirable flavor compounds in microalgae [108]. These compounds primarily originate from lipid oxidation processes, especially the degradation of polyunsaturated fatty acids (PUFAs), as well as from carotenoid breakdown during harvesting, drying, and storage. Established approaches for the reduction of these off-flavor compounds include deaeration and heating, enzymatic treatments, fermentative processes to modify volatile profiles, and adsorptive techniques for the removal of odor-active molecules [109]. However, these methods often introduce trade-offs in terms of protein yield, functionality, or process complexity. As another alternative, microencapsulation emerges as a promising strategy to retain bioactivity and mask off-flavors, particularly for *Arthrospira*. Consumer acceptance is highest when visible improvements (e.g., enhanced color or texture) are accompanied by minimal sensory disruption, suggesting that tailored formulations are key to market viability.

Another important aspect of single-cell protein integration is the relatively high RNA content in microalgae, typically between 4 and 6% of dry cell weight [110]. This factor is often overlooked in food-oriented studies. Elevated intake of nucleic acids may lead to potential health concerns, such as increased uric acid levels and an associated risk of hyperuricemia, and is, in general, a problem with single-cell-derived food. Nucleic acid content can be reduced by enzymatic RNA degradation (e.g., endonuclease treatment), thermal processing, or combined extraction–purification strategies [111]. However, such mitigation approaches remain underrepresented in the literature for microalgae, and their impact on protein yield, functionality, and process economics has not yet been systematically addressed.

A systematic query of the Global New Products Database (GNPD) identified a total of 22,849 food products and beverages launched between 2020 and 2024 that contain microalgal-based ingredients [112]. This analysis specifically included ingredients such as *Arthrospira*, algae extracts, algae protein, and algae collagen, while deliberately excluding products containing macroalgae (e.g., nori, wakame, kombu, Irish moss, and related species) to ensure a focused assessment of microalgal applications. To contextualize these findings, a parallel GNPD search for products containing any form of plant protein launched between 2020 and 2024 yielded 18,961 results. The top five product categories incorporating microalgal ingredients were as follows: Desserts and Ice Cream (27.4%), Dairy (15.6%), Processed Fish, Meat and Egg Products (10.4%), Sugar and Gum Confectionery (9.7%), and Meals and Meal Centers (8.1%) [112]. Amongst the microalgal-based ingredients, *Arthrospira* was by far the most frequently used, appearing in various forms such as *Arthrospira* concentrate (14.5%), *Arthrospira* extract (4.1%), or generically labeled as algae (4.8%) or algae extract (1.0%) [112].

**Figure 2:**
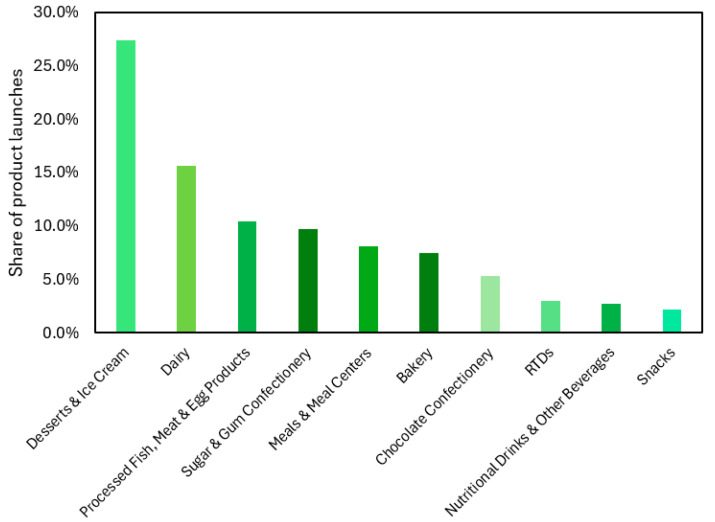
Microalgal-based product launches in food and drinks (2020–2024) by product categories (*n* = 22,849) abbreviation: RTD: Ready to Drink.

**Figure 3:**
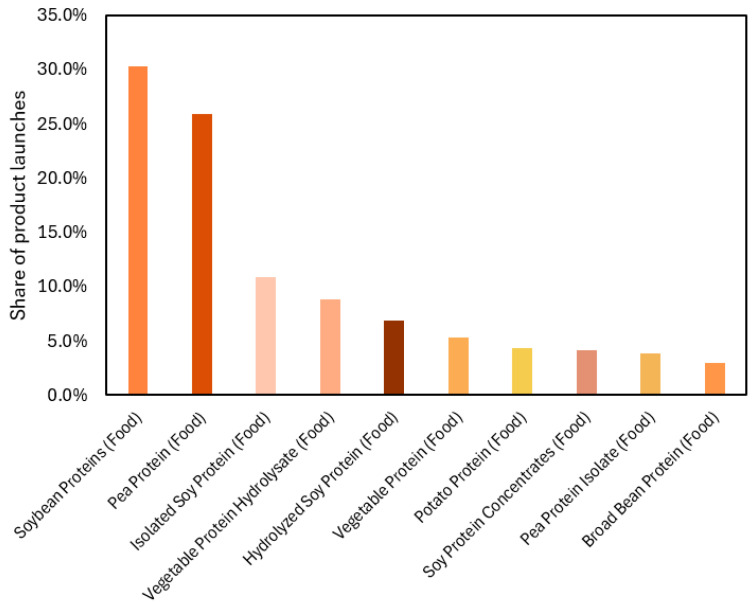
Plant protein product launches in food and drinks (2020–2024) by ingredients (*n* = 18,961).

A more specific search targeting microalgal protein as an ingredient resulted in only three product launches during the same period. All of these products were developed by the Czech company Heaven Labs under the brand Mana, including two protein powder drinks and one plant-based burger. In each case, algae protein was part of a composite plant protein blend, alongside common sources such as soy, pea, oat, hemp, and rice proteins [112]. These results indicate that microalgal ingredients are gaining relevance in new product development, especially in food categories such as desserts and dairy alternatives, whereas algae protein itself remains a niche ingredient. Figure 2 shows food and drink products into which microalgae have been integrated, whereas Figure 3 presents plant-based protein products launched in the food and beverage sector.

## 4. Value-Added Potential of Microalgal Protein Concentrates and Isolates

Processing algae into protein concentrates or isolates can mitigate limitations such as sensorial and functional attributes, increase their appeal, and improve both digestibility and nutritional value and formulation flexibility, while enabling targeted tailoring of protein properties [113,114]. Understanding protein digestibility is very important for the design of novel food matrices. Digestibility depends on factors such as strain, cell wall disruption, and processing methods. Isolates improve digestibility and protein uptake by removing components that hinder digestion, such as rigid cell walls [115]. Standard In Vitro Protein Digestibility (IVPD) models, like the Boisen and Fernández method, assess digestibility by measuring enzymatic hydrolysis, while in vivo studies offer more precise insights but raise cost and ethical concerns [116,117]. Future advancements in computational digestion models [118] may refine protein quality assessments, supporting the broader application of microalgae as a sustainable protein source in novel food matrices. Other important metrics involve the Indispensable Amino Acid Score (IAAS), Protein Digestibility-Corrected Amino Acid Score (PDCAAS), and Digestible Indispensable Amino Acid Score (DIAAS) [119]. These are methods used to assess protein quality based on the content and availability of essential amino acids. While the IAAS considers only the essential amino acid profile relative to a reference pattern, the PDCAAS additionally accounts for the overall digestibility of the protein, and the more recent DIAAS evaluates the digestibility of individual indispensable amino acids at the ileum, providing a more accurate measure of biological value [119]. Currently, the Food and Agriculture Organization of the United Nations (FAO) recommends DIAAS as the preferred method for measuring protein quality [120]. These parameters should be carefully evaluated when assessing the protein profiles of novel microalgal strains or even those currently utilized by the food industry, as they facilitate the comparison of protein quality across different species.

Beyond nutritional aspects, algal proteins offer key techno-functional properties, including solubility, emulsification, foaming, and gelling that are crucial for food quality, structure, and stability, as demonstrated in several studies in Table 4. Protein solubility is a key factor in enabling a broad range of downstream applications. However, it is often challenging to achieve with conventional plant-based protein sources, which require harsh processing conditions to enable solubility. In contrast, several microalgal species contain high concentrations of soluble proteins over a wide pH range. However, solubility is dependent on the environmental physicochemical conditions, which can vary substantially across different food matrices, thereby posing significant challenges for food applications and requiring tailored processing methods. Beyond solubility, microalgal proteins exhibit emulsifying properties that enable oil-water stabilization, which is important in food products, such as mayonnaise, while foaming and gelling properties are essential for products, such as mousses, gels, or pastries. Moreover, solid protein powders can be structured into fibers, acting as meat analogs. Tailoring these properties for various food matrices enhances the applicability of algae-based ingredients in mainstream food production, mimicking animal-based food products. Table 4 presents various studies that tested different technofunctional properties of microalgal protein.

## 5. Cultivation of Microalgae for Protein Production

### 5.1. Phototrophic Cultivation: Challenges and Constraints

The feasibility of microalgae as protein sources for food applications ultimately depends on their scalable cultivation and achievable biomass productivity [10]. Phototrophic cultivation of microalgae differs fundamentally from that of typical heterotrophically produced microorganisms [128]. For commercial applications, some microalgae capable of utilizing organic carbon sources can preferably be produced heterotrophically, as this yields high biomass concentrations and facilitates scaling as well as downstream processing [129]. For instance, *Chlorella sorokiniana* can be produced with a final biomass concentration of more than 271 g/L [130]. However, the carbon source in this process often depends on agricultural feedstock and, thus, limits long-term sustainability, highlighting the need for agriculture-independent cultivation strategies. Therefore, this section of the review specifically focuses on phototrophic cultivation, in which light serves as the primary energy source and must be supplied efficiently to the culture medium. This fundamental requirement has strongly shaped the design of cultivation systems commonly used in algal biotechnology. As a result, biomass productivities and yields remain significantly lower than in heterotrophic systems, reinforcing the necessity for ongoing research to support widespread application [129]. Meeting biomass demand—for sufficient protein supply—requires upscaling these cultivation systems to vast areas or space, which presents a significant challenge given the approaches summarized in this chapter.

In phototrophic microalgae cultivation, two methods are conventional on an industrial scale: open and closed pond cultivation [129]. Key requirements for successful cultivation are confirmed in both approaches. Gas transfer from the atmosphere to the medium is one of the foremost limiting criteria, followed by bringing light into an aqueous environment adequately. CO_2_ as the primary carbon source and light as the primary energy source must be provided optimally to the culture in order to achieve and maintain optimal growth during autotrophic cultivation [131]. The most common light sources are natural sunlight or artificial light sources such as LED [132]. Light supply has influenced the design of photobioreactors to ensure high surface exposure and efficient light distribution, typically achieved through narrow diameters and large illuminated areas. These reactors are often made of transparent materials such as glass or clear plastics to maximize light penetration and photosynthetic efficiency. Effective mixing is a critical aspect of microalgal cultivation, facilitating the uniform distribution of cells and light, regulating temperature and metabolite levels, and enhancing gas transfer within the system. Mixing typically involves controlled airflow or mechanical agitation systems. In large-scale production, a certain degree of turbulence is advantageous to promote the rapid circulation of microalgal cells between the light and dark zones of the reactor in order to achieve homogenous illumination. However, it is imperative to exercise caution as excessive turbulence resulting from mechanical agitation or air bubbles can induce shear forces, thereby causing damage to microalgae. The optimal level of turbulence, beyond which cell mortality is observed, is dependent upon the specific strain of microalgae and necessitates meticulous investigation and strain specific optimization to prevent any compromise in productivity.

As with any microorganism cultivation, optimal working temperatures are linked to the physiological range of the specific strain. Contamination risk is another critical factor that could be addressed by selective growth conditions, chemical treatments, and co-cultivation of other probiotic microbes for robust polycultures [133]. Soluble products that are released by algae into the medium can be a limiting factor, as such compounds may accumulate in the culture, thus inhibiting algae growth [134]. Maximizing bioproduct yields requires simple, robust, and cost-effective system design and operation, given the considerable challenges associated with phototrophic algae biotechnology, as discussed above.

### 5.2. Open Systems

Open systems are simple and have been used for a long time. Typical cultivation modes in open ponds include tanks, raceway ponds, circular ponds, and open thin-layer cascade photobioreactors [135,136].

The most widely disseminated and cost-effective method for cultivating microalgae is the open raceway pond (ORP) system, which consists of ponds with depths ranging from 0.2 to 0.5 m [137] and includes paddle agitation and mixing equipment [138]. One of the main challenges in outdoor algae cultivation is biotic contamination—such as bacteria, fungi, amoebae, insects, and others—caused by exposure to the atmosphere [139]. These contaminants pose significant problems, especially in food production. The production yield is also very limited and depends on weather and climate conditions, with typical productivities rarely exceeding 12.9 g/m^2^/d [140]. Typical final cell concentrations obtained from these cultivation modes are summarized in Table 5. In contrast, open thin layer cascade reactors have demonstrated high cell densities greater than 30 g/L in a time span of 14 days and a biomass productivity of 14.9−21.3 g/m^2^/d, exceeding ranges typically reported for open ponds [136].

### 5.3. Closed Systems

**Figure 4:**
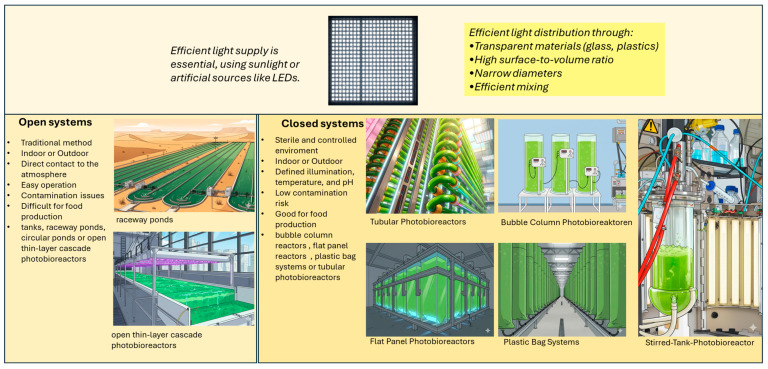
Overview of various microalgal cultivation modes and corresponding pros and cons.

For biotechnological and especially food purposes, photoautotrophic algae can also be cultivated in closed systems in sterile and controlled environments. Such systems allow precise control over parameters like illumination, temperature, and pH, enhancing microalgal growth and productivity. This control also helps to meet Good Manufacturing Practice (GMP) requirements to produce bioproducts for human use. Prominent PBRs in this field are bubble column reactors, flat panel reactors, plastic bag systems, or tubular photobioreactors due to their ease of application [151]. Each design varies in its specific approach to surface area utilization and scalability [151]. These closed cultivation systems are designed to enhance microalgal production through optimized light exposure and controlled environments. Productivities are difficult to compare with open systems since titers are often published in volumetric values. Daily productivities vary in literature, with typical values ranging between 0.3 and 1.47 g/L/d for *Chlorella* [152] and 0.079–1.319 g/L/d for *Arthrospira* reported for closed systems [153]. In addition to open ponds, Table 5 also includes production performance of closed systems. Aberu et al. dedicated a review to this topic, listing several microalgal species and reporting productivities as well as final cell concentrations under different cultivation modes (heterotrophic and autotrophic) [154]. Figure 4 provides an overview of the different cultivation systems used in microalgal biotechnology.

### 5.4. Cultivation-Strategies to Improve Biomass Productivity and Protein Content

Optimization of protein production in microalgae can be achieved by adjusting culture conditions such as medium composition, carbon-to-nitrogen (C/N) ratios [155], pH adjustments, light conditions, and considering the growth stage during cell harvest [37]. In a study by Cao et al., BG11 medium for *Chlorella sorokiniana* cultivation was optimized, resulting in tripling the protein content, highlighting the significance of fine-tuning medium composition [156]. Carbon-to-nitrogen ratio is particularly critical in optimizing protein production. Renyuan Jiang et al. investigated the effects of different ammonia concentrations and pH levels on *Chlorella vulgaris*. They found that higher ammonia concentrations increased protein content but reduced growth performance, while pH had a significant impact on both growth and overall protein yield [157]. Nitrate and iron, as macro- and micro-nutrients, respectively, have been observed to be the limiting compounds for different species, with copper being essential for growth in marine media [158]. To fully understand cultivation performance and protein accumulation, it is necessary to correlate medium depletion to the growth pattern.

Cultivation mode is another key point of optimization. As typical batch cultivation shows biomass contents lower than 10 g/L/week (dcw), as highlighted in Table 5, it might be promising to test multiple cultivation strategies, such as fed-batch or modes of continuous production. Key challenges with low cell densities remain, which need to be overcome, for instance, by applying continuous production systems with medium recycling without energy-demanding cell recovery technologies. Especially, continuous production allows the production of young biomass, which might have another impact on protein yields. To the best of our knowledge, only limited research has been conducted in this area, representing an important research gap in microalgal biotechnology. Muys et al. emphasized the role of continuous cultivation in optimizing protein and essential amino acid (EAA) productivity [62]. Interestingly, the highest protein productivity did not necessarily correlate with the highest biomass productivity. In their study, raceway ponds yielded higher outputs for *Chlorella vulgaris*, whereas *Limnospira indica* performed better in closed photobioreactors, reaching protein productivities of 300 mg/L/day and EAA yields of 33 mg/L/day. Continuous operation under constant light improved process control and increased EAA yields by up to 50%, highlighting its potential for high-quality, large-scale protein production. Koruyucu et al. performed continuous cultivation experiments in an open thin-layer cascade photobioreactor with *Microchloropsis salina*, reaching areal productivities of 35 g/m^2^/d, which is comparably high to other open pond systems, performed in batch mode [159].

## 6. Downstream Processing of Microalgal Biomass

Several reviews already describe the downstream processing of microalgae to obtain protein production [160,161]. This review aims to provide a concise overview of the methods applied during downstream processing of microalgal biomass to extract proteins, highlighting their advantages and limitations to better contextualize current challenges. These challenges are among the key reasons why microalgal proteins have not yet achieved widespread commercial adoption.

### 6.1. Cell Harvesting Methods

Following cultivation, efficient biomass harvesting represents the first critical downstream step determining the feasibility of microalgal protein recovery. Handling large culture volumes and low cell concentrations, typical of phototrophic cultivation, is challenging, making process intensification a critical step in phototrophic algae biotechnology. These techniques include centrifugation, flocculation, sedimentation, flotation, and filtration. Table 6 summarizes the principles, advantages, and disadvantages of different methods used for microalgal cell harvesting. Evaluating the efficiency and associated costs of these methods is essential for identifying the most suitable approaches for industrial application [151]. For protein extraction, wet biomass is generally preferred over dried material, as drying processes can significantly affect protein yield and functionality [162].

In general, individual harvesting methods exhibit distinct advantages and limitations, and no single technology simultaneously achieves high efficiency, low cost, and low energy consumption. For example, Zhang et al. demonstrated that the combined process of flocculation and filtration can complement each other, and the cost can be significantly reduced from 0.206 $/kg (filtration without flocculation assistance) to 0.139 $/kg [183]. Overall, efficient and sustainable microalgal harvesting requires the strategic integration of complementary technologies based on production scale, strain characteristics, and economic and energetic feasibility.

### 6.2. Cell Disruption Methods

Following cell harvesting and concentration, cell disruption is required to release intracellular proteins into the surrounding medium. The composition and rigidity of microalgal cell walls vary substantially among species, often posing significant barriers to efficient disruption, eventually necessitating substantial energy input [184]. Therefore, cell disruption emerges as an important step in the downstream process that strongly influences protein recovery efficiency and integrity. Typically, the choice of cell disruption method depends on the cell wall structure of the algal species, product location, cell size, and the amount of energy applied. Protein recovery particularly requires methods with lower energy input to avoid denaturation or proteolytic digestion, which would have implications on the technofunctional properties of the proteins. Disruption methods are divided into physical/mechanical methods (e.g., bead milling, high-speed or high-pressure homogenization, and ultrasound, etc.) and non-mechanical methods (e.g., chemical or enzymatic treatment) [185]. Table 7 provides a comprehensive overview of commonly applied methods for microalgal cell disruption and summarizes their respective advantages and limitations. Overall, no single cell disruption technique simultaneously achieves high efficiency, low energy demand, high selectivity, and gentle processing conditions. Mechanical methods are generally effective and scalable but often involve high energy input and limited selectivity, whereas non-mechanical approaches can offer milder conditions but suffer from slow processing rates, higher costs, or scalability constraints.

### 6.3. Protein Extraction Method

Protein concentrates and isolates typically exhibit high protein content, which necessitates the removal of undesired compounds such as carbohydrates, lipids, flavor-active compounds, and pigments. Selective enrichment of proteins in the final downstream stages is therefore critical yet technically demanding. Microalgal proteins comprise both soluble and insoluble fractions. However, many studies primarily focus on the recovery of soluble proteins due to their relevant techno-functionalities, as demonstrated in Table 4. However, common extraction methods can affect final solubility, as the isolation conditions may alter protein-related properties such as solubility and other technofunctionalities by altering the environment’s pH and hydrophobicity.

Common extraction methods involve alkaline/acidic treatment, whereby alkaline conditions increase protein solubility and acidic conditions lead to precipitation, typically in pH ranges where most proteins have their isoelectric point. In *Chlorella*, the majority of soluble proteins have pI-values in a range of 4.0–5.5 [220], and dissolved *Arthrospira* proteins tend to precipitate around pH 4 [221]. Proteins can also be precipitated by the salting-out strategy, e.g., by adding salts such as ammonium sulfate. Protein content can also be enriched by organic solvent treatment such as ethanol and acetone. Alternatively, membrane-based processes (e.g., micro-, ultra-, or nanofiltration, reverse osmosis) can be used for selective separation and concentration of proteins while retaining biological activity. Concentrating proteins in an aqueous multiphase system can separate microalgal components into different aqueous phases, which are highly favorable for maintaining protein integrity.

**Figure 5:**
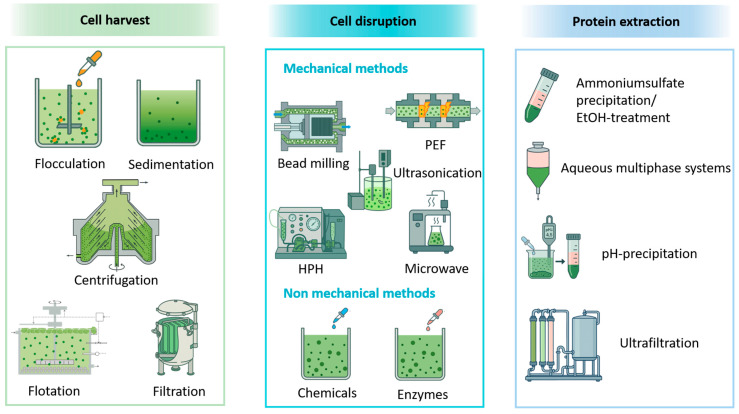
Overview of typical downstream process-steps necessary for microalgae protein isolation. Abbreviations: PEF: pulsed electric field, HPH: high-pressure homogenization.

Nevertheless, many extraction methodologies could introduce substances that must be removed to meet food-grade standards [222]. This requires further purification steps such as centrifugation, filtration, ultrafiltration, chromatography, or drying, which could have a substantial impact on production costs and protein integrity. To improve the accessibility and commercial viability of algal proteins, cost-efficient processes are required that maintain key techno-functional properties such as solubility while being scalable and compatible with food industry requirements. Table 8 summarizes representative extraction and enrichment methods reported for food-grade microalgal products. Figure 5 provides an overview of downstream processing technologies relevant to microalgal protein production.

### 6.4. Protein Quantification Methods

Isolating proteins from microalgae prior to protein quantification may be essential to eliminate interfering substances and could reduce variability between methods, potentially providing a more accurate protein content. Kjeldahl, Lowry, or Bradford assays are the most commonly used methods. However, these can yield inconsistent results due to interactions with non-protein compounds. Total nitrogen methods often overestimate protein levels because microalgae contain significant non-protein nitrogen, varying by species and conditions. For example, using a standard conversion factor (kp = 6.25) [235] can result in overestimated protein values, which is why more accurate species-specific factors (~4.78–5.3) have been proposed [236]. Colorimetric assays vary in accuracy and measure soluble proteins: the Lowry method often overestimates protein content due to interference with phenolic compounds and other reducing agents, while the Bradford method may underestimate it in samples low in arginine moieties [237,238]. More precise techniques like amino acid analysis by LC-MS detection reveal these discrepancies. In a study conducted by Weber et al., the soluble protein content was measured using both the Lowry and Bradford assays, and results revealed substantial differences between the two methods; the Lowry assay measured up to three times higher protein concentrations than that of the Bradford assay in the same samples [239]. The use of different quantification approaches across the literature complicates the accurate assessment of the true protein production potential of microalgae and hinders meaningful comparisons between studies. Therefore, for the successful application of microalgal proteins in food systems, the implementation of reliable and standardized quantification methods is essential to ensure consistency and comparability.

## 7. Protein Processing Methods for Tailoring Food Properties

By modifying the amino acid side chains or higher-order structures, it is possible to obtain alternative or “tailored” protein functional properties. This section explores the most common methods to modify protein properties. As microalgal protein becomes increasingly available, such modification approaches, typical for native isolated plant-based protein fractions, may be essential to enable its use as a versatile ingredient in a wide range of processed food products. A wide variety of physical and chemical methods are available to modify the structure of proteins and thus control their functional properties. Typically, a combination of different modification techniques is employed for synergistic effects. Challenges including scalability (cost-effective production) and sustainability (green and eco-friendly) are actively explored and addressed.

### 7.1. Chemical Modification

Chemical modifications alter protein properties by adding, removing, or modifying functional groups of amino acids, thereby influencing the protein structure. Recent studies that have focused on sustainable and food-safe methods adopted: (1) Succinylation: a post-translational modification in which succinyl groups are added to the side chains of lysine [240]. (2) Glycosylation involves adding carbohydrate moieties to proteins [241]. (3) And, acetylation involves the addition of acetyl groups to lysine residues [242]. These methods improve solubility, digestibility, thermal stability, oil and water absorption capacity, and foam stability of proteins. However, alongside several desired properties, undesired effects—such as reduced foaming capacity, lower digestibility, or overall impairment of protein functionality—may also be observed.

### 7.2. Heating

Heat-induced (partial) denaturation exposes hydrophobic groups, thereby altering functional properties. Heating treatments can improve gelation properties, hardness, and water-holding capacity [243]. Various heating methods have been explored for their effects on protein functionality: (1) Microwave heating is fast and efficient but may result in uneven heat distribution. (2) Ohmic heating uses electricity to generate heat directly within the product. Here, an electric current passes through the sample, and the resistance within the sample generates heat evenly and quickly [240]. However, associated challenges may arise due to electrode corrosion or uneven heat distribution, which can lead to deviations in achieving the desired functionalities.

### 7.3. Enzymatic Treatment

Enzymes catalyze specific and targeted modifications under mild conditions, offering precise control over protein properties: (1) Transglutaminases form covalent bonds between glutamine and lysine residues, resulting in macromolecular assemblies and stable complexes (protein crosslinking) [244,245]. This treatment enhances gel strength and water-holding capacity. (2) Proteases break peptide bonds, exposing side chains, thereby altering functional properties [246,247]. This modification improves the digestibility, solubility and emulsifying properties of proteins. (3) Protein-glutaminases deamidate glutamine residues, altering protein charge and hydrophobicity, also improving solubility and emulsifying properties of target proteins [248].

### 7.4. Physical and Mechanical Methods

**Figure 6:**
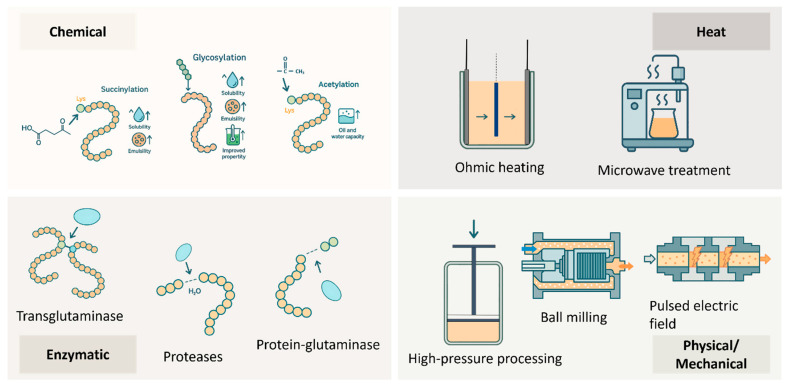
Typical protein processing methods aimed at improving protein functionality in food.

Various physical and mechanical methods can be employed to alter and improve the physicochemical properties, functions, and applications of proteins and protein isolates. These include: (1) High-pressure processing is a non-thermal method that can reduce particle size and improve solubility and emulsifying properties but is associated with high costs [249,250]. (2) Ball milling uses steel or rubber balls for grinding, reducing particle size and increasing surface area, but heat generated during the process might affect the protein structure in an undesired way, leading to denaturation [251]. (3) Ultrasonication employs high-frequency sound waves to disrupt protein structures and enhance techno-functionalities such as solubility, foaming, emulsification, and gelation [252]. (4) In high-moisture extrusion cooking, proteins are aligned under high temperature, pressure, and moisture in an extruder to form a fibrous, meat-like structure [253]. Figure 6 provides an overview of different protein processing methodologies available for tailoring protein function. Various examples related to protein modification are summarized in Table 9.

## 8. Economic Feasibility of Microalgal Protein Isolates

The production cost of dried microalgal biomass for food purposes is currently estimated at approximately 10–50 USD/kg, whereas whey and soybean proteins are available at substantially lower costs of around 5–10 USD/kg and 1–2 USD/kg, respectively [210]. Biomass production costs are still a bottleneck that needs to be urgently addressed by advancing cultivation technologies. Open pond systems represent the most cost-effective approach for microalgal biomass production, with reported costs lower than 5 USD/kg [264,265]. In contrast, closed photobioreactor systems provide higher process control and product quality and enable the production of high-protein biomass but typically involve substantially higher production costs depending on scale and operational conditions compared to open pond systems. Biomass that was produced via low-cost cultivation methods can serve as a viable basis for protein extraction processes aimed at producing economically feasible protein isolates. Downstream processing also has a significant impact on overall production costs. The higher the desired protein purity, the more complex and cost-intensive the downstream processing becomes, which is similarly observed for protein isolates from other sources on the market. This remains a challenging target given current processing technologies. Notably, a study by Tzachor et al. estimated a land requirement of 0.0378 m^2^/per kg of *Arthrospira* biomass. Considering the measured protein content of 27.2%, this corresponds to 0.14 m^2^ per kg protein [266]. In contrast, the areal demand for beef, pork, and chicken ranges between 144 and 258, 47 and 64, and 42 and 52 m^2^/kg protein, respectively [267]. These numbers demonstrate the added value of proteins when sourced from microalgal cultivations that require minimal land space. Microalgae offer advantages in terms of land independence, high productivity potential, and the ability to produce high-value functional proteins under controlled conditions, which may justify their use in premium or functional food applications rather than bulk protein markets. However, this economic constraint could be substantially reduced if significant scientific and technological advancements in microalgae cultivation and processing are achieved.

## 9. EU Policy Recommendations

The global population is projected to increase to 9.7 billion in 2050 [268], with strong regional disparities. Sub-Saharan African countries are expected to grow at twice the rate of the global average, with India surpassing China in population. The 17 UN Sustainable Development Goals (SDGs) aim to address global challenges like overpopulation, climate change, poverty, illiteracy, and food insecurity through interconnected targets. “Zero Hunger” (SDG No. 2) is key to achieving goals like health, education, climate action, and sustainable development [269]. Malnutrition and malnourishment are urgently to be addressed across global populations, requiring major investments in agriculture, food systems, and supply chains. Food security depends not only on increased production but also on supporting smallholder farmers, sustainable practices, and land restoration [270]. Climate change further complicates these efforts due to increasing events of extreme weather like droughts or floods. Furthermore, restoring agricultural land is vital for biodiversity, water quality, and natural carbon sinks [271]. Alongside improving traditional crops, alternative/complementary food systems such as microalgae offer promising solutions independent of arable land. These could especially benefit urban regions with scarce farmland—such as city-states in Asia, desert cities, or countries that heavily rely on food imports. Photobioreactors, which require minimal land area, can operate under controlled conditions, offering a viable solution for regions facing constraints in traditional food production [272]. Cultivation medium recycling allows for the recovery of water and dissolved nutrients, thereby reducing the demand for fresh fertilizers and minimizing nutrient losses common in large-scale agriculture.

The EU has increasingly acknowledged the potential of algae as a sustainable resource within its strategic policy frameworks. Algae were explicitly integrated into the “European Green Deal” (2019) [273] and subsequently reflected in the “Farm to Fork Strategy” (2020) [274], which emphasizes the need for resilient, climate-neutral food systems. The “Strategic Guidelines for a More Sustainable and Competitive EU Aquaculture” (2021) [275] further highlights algae as a promising alternative protein source. In 2022, the European Commission published the Action Plan: “Towards a Strong and Sustainable EU Algae Sector” [275], outlining a comprehensive roadmap to foster algae-related innovation and market growth. Market projections indicate a compound annual growth rate (CAGR) of 6.4% for *Chlorella* and 8.7% for *Arthrospira*, underlining the sector’s economic relevance. To accelerate the development of the European algae sector, the European Commission has launched several funding programs and initiatives, including EU4Algae, Horizon Europe, the Circular Bio-Based Europe Joint Undertaking, and related funding programs. [275,276,277,278]. The action plan “Towards a Strong and Sustainable EU Algae Sector” (2022) defines 23 measures across governance, innovation, market development, and public awareness [275]. This review aligns with key objectives of the action plan, particularly in relation to standardization of methods, nutrient extraction from algal biomass, advances in processing and production technologies, and strategies to improve market acceptance of algae-based products.

While these EU strategies address algae as a broad category—within which macroalgae already have an established history of food use—microalgae remain a comparatively underdeveloped food resource, requiring dedicated research, technological maturation, and regulatory clarification to enable their large-scale integration into European food systems.

In order to be economically viable, the whole algal biomass must be utilized through integrated biorefinery concepts aligned with circular bioeconomy and waste-valorization principles, ensuring maximum value extraction, resource efficiency, and the conversion of all biomass fractions into multiple high-value products across different sectors—an approach that still requires substantial research and innovation efforts [279]. Within this framework, sustainable biomass production is a critical determinant of overall system performance, with the carbon source representing a key limiting factor. At present, most commercially available CO_2_ originates from fossil fuels or industrial waste streams, which undermines sustainability objectives. It is therefore essential to develop and implement strategies to secure alternative, sustainable carbon sources. In particular, atmospheric CO_2_ capture and the valorization of biogenic waste streams, such as those from biogas plants, are key to ensuring the long-term viability and climate neutrality of algae-based protein production.

Advancing microalgal protein research could enable the emergence of a new European industry centered on microalgae as a widely applicable protein source. The worldwide demand for vegan proteins is steadily increasing, with an estimated increase of 7% CAGR from 12.5 billion US dollars in 2025 to 24.5 billion US dollars in 2035 due to growing consumer preference for plant-based or alternative sustainable food products [280]. Most plant-based proteins are derived from sources like soy, wheat, and peas. These typical plant-based resources often face challenges in meeting the nutritional profile or desired techno-functionality known from animal-based sources [281]. Due to their favorable techno-functional properties and balanced amino acid profile, algal proteins could serve as promising alternatives to conventional animal or plant-based protein sources in food. Further research is needed to define the necessary steps for the targeted and scalable processing of microalgal protein isolates, as their effective incorporation into complex food systems remains largely unexplored, representing a major research gap that urgently needs to be addressed. Subsequent processing of proteins might be a necessary step to achieve the defined function in food. From an economic and developmental perspective, initiating industrial-scale production using heterotrophically cultivated microalgal biomass and well-established protein isolation methods could facilitate rapid implementation of downstream processing and protein integration into food applications. This approach may facilitate faster market entry while enabling subsequent research and infrastructure development for more sustainable phototrophic cultivation systems and cost reduction for necessary downstream processes towards the protein isolate.

As stated earlier, the EU Novel Food Regulation (EU) 2015/2283 [45] governs the authorization of foods and ingredients that were not significantly consumed within the EU before 15 May 1997. This regulation aims to ensure the safety of novel foods for human consumption and the accuracy of their labeling prior to market entry. With the expected increase in the number and diversity of microalgal-derived food products in the coming years, continuous research on their composition, bioavailability, and physiological effects will be essential to evaluate potential health benefits, allergenic risks, and metabolic impacts. Such research should proceed in parallel with product development to ensure consumer safety while enabling efficient market introduction.

Cultivation of microalgae on wastewater is a quite promising research field for sustainable biomass production, due to a low-cost nutrient source enabling circular bioeconomy concepts [282,283]. However, a critical issue in wastewater-coupled microalgae cultivation is the potential accumulation of contaminants in the produced biomass. This aspect is frequently overlooked in studies focusing on food applications. Particular concerns include the uptake of heavy metals such as cadmium, lead, and mercury, as well as antibiotic residues and microplastics. Despite their relevance for food safety and consumer acceptance, these contaminants are still rarely investigated in food-oriented microalgae studies. Given the strong biosorptive and bioaccumulative capacity of microalgae, there is a realistic risk that such compounds are concentrated within the biomass and may subsequently be present in the food product. Despite the relevance of this issue for human and animal health, food safety considerations are often not integrated into process design or techno-economic assessments. In particular, regulatory frameworks such as those provided by the EFSA, including contaminant thresholds and safety evaluation criteria for Novel Foods, are frequently not explicitly addressed in the context of wastewater-derived microalgal biomass. This disconnect between process development and regulatory safety assessment represents a significant barrier to the direct application of such systems in food markets.

Furthermore, safety and regulatory considerations remain important challenges for the incorporation of microalgal metabolites into food products. Depending on the species and metabolite, concerns include the presence of algal toxins, contamination with cyanobacterial toxins, and potential allergenicity [284]. While food-grade microalgae generally exhibit a favorable safety profile, documented cases of allergic reactions to Arthrospira (spirulina) and other edible algae have been reported [285,286], and allergenicity data for many novel microalgal metabolites remain scarce. Therefore, comprehensive toxicological and allergenicity assessments are required before the commercialization of novel microalgal ingredients.

**Figure 7:**
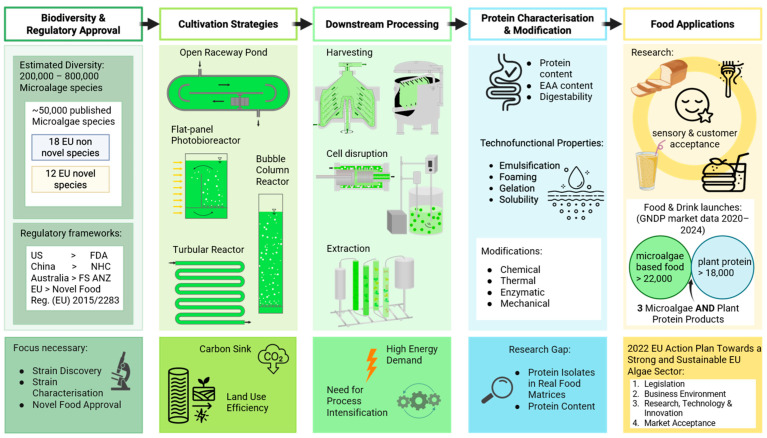
Overview of the entire protein production pipeline, highlighting the different stages where targeted research and funding efforts are required to ensure the successful market introduction of microalgal protein.

As shown in this review, research on microalgal proteins is inherently complex and requires coordinated, interdisciplinary approaches in which different fields exchange knowledge and complement each other. Political commitment and support are crucial to ensure that research strategies can be effectively implemented. We put forth our policy recommendations in Table 10, aimed at advancing microalgal protein implementation in the food sector, as well as their respective expected results and acting entity. The successful establishment of a microalgal protein industry could not only meet the growing demand for sustainable food solutions but also create new employment opportunities in the biotech and food sector. Moreover, such an industry could help transfer technologies to food-insecure regions, especially in regions with limited resources and research infrastructure. Such a development could contribute to addressing global nutrition challenges and support progress towards achieving the SDGs. Figure 7 shows an overview of the whole protein production pipeline.

## 10. Conclusions

Microalgae are a promising sustainable protein source with the potential to contribute to future food security while reducing the environmental impacts associated with conventional animal-based proteins. Their favorable amino acid profile, versatile techno-functional properties, and low land requirements make them attractive ingredients for a wide range of food applications. Despite these advantages, several scientific, technological, economic, and regulatory challenges continue to limit large-scale implementation. Biomass production costs remain high compared to conventional protein sources, and downstream processing represents a major economic bottleneck, particularly when high-purity protein isolates are required. The integration of microalgal proteins into complex food matrices and the development of targeted processing strategies therefore require further investigation. Successful commercialization of microalgal proteins will depend on coordinated progress across the entire production chain, including cultivation, harvesting, protein extraction, biorefinery integration, protein purification, functional characterization, food formulation, and regulatory assessment. Particular attention should be given to food safety aspects, especially when alternative cultivation concepts such as wastewater-based systems are considered. From a policy perspective, continued public investment, interdisciplinary collaboration, and supportive regulatory frameworks are essential to accelerate technological development and market adoption. The EU has already established important strategic initiatives to promote algae-based innovation; however, dedicated efforts are still required to bridge existing research gaps, improve economic feasibility, and strengthen consumer acceptance. With continued advances in cultivation technologies, downstream processing, and product development, microalgae have the potential to become a key component of a resilient, resource-efficient, and climate-friendly global complementary protein supply.

## Figures and Tables

**Table 1:** Microalgae with the status of non-novel food as listed in the EU Novel Food Status Catalog [48].

Not Novel			
Taxonomy/Species	Status in the Catalog (Authorization)	Reported Protein Content	Reports on Amino Acid Profile
*Aphanizomenon flosauae*	Not novel in food supplements	60–70% [42]	[49]
*Arthrospira* *A. platensis*	Not novel in food	55–70% [50]	[50]
*Auxenochlorella* *A. protothecoides* *A. pyrenoidosa*	Not novel in food Not novel in food	62–68% [51]	[52] (Studies performed in self-generated Auxenochlorella mutants)
*Chlorella* *C. pyrenoidosa* *C. sorokiniana* *C. vulgaris*	Not novel in food Not novel in food Not novel in food	44% [53] 17–43% [53] 27–40% [53]	[53]
*Dunaliella* *D. salina*	Not novel in food supplements	19–41% [54]	[55]
*Graesiella* *G. emersonii*	Not novel in food	66% [56]	[57]
*Haematococcus* *H. lacustris*	Not novel in food supplements (Astaxanthin-rich oleoresin from Haematococcus pluvialis is authorized novel food)	25–60% [58]	[59]
*Heterochlorella* *H. luteoviridis*	Not novel in food	29% [60]	-
*Jaagichlorella* *(new name of H. luteoviridis)* *J. luteoviridis*	Not novel in food	29% [60]	*-*
*Limnospira* *L. fusiformis* *L. Indica* *L. maxima*	Not novel in food Not novel in food Not novel in food	60–70% [61] - 44–58% [62] 45.50% [63]	[62,63]
*Parachlorella* *P. kessleri*	Not novel in food	19–54% [64]	-
*Scenedesmus* *S. vacuolatus*	Not novel in food	45% [65]	-
*Spirulina* *S. major*	Not novel in food	57% [66]	-

**Table 2:** Microalgae with the status novel food as listed in the EU Novel food Status Catalog [48].

Novel			
Taxonomy/Species	Status in the Catalog (Authorization) Yes/No	Reported Protein Content	Reports on Amino Acid Profile
*Chlamydomonas* *C. reinhardtii*	No (authorized pursuant to the requirement)	47% [67]	[67]
*Desmodesmus* *D. communis* *D. subspicatus*	No (authorized pursuant to the requirement) No (authorized pursuant to the requirement)	35% [68] 12–27% [69]	[68]
*Schizochytrium* *Schizochytrium* sp. Docosahexaenoic acid (DHA) and Eicosapentaenoic acid (EPA) ethyl esters oil from *Schizochytrium* sp.	No (authorized pursuant to the requirement)	11% [70]	[70]
*Euglena* *E. gracilis*	No (authorized pursuant to the requirement) Dried biomass is authorized as novel food)	47–60% [71]	[72]
*Odontella* *Odontella aurita*	Yes, Authorized novel food	9–30% [73]	-
*Scenedesmus* *S. acutus* *S. quadricauda*	No (authorized pursuant to the requirement)	53% [74] 65% [75]	[76]
*Tetradesmus* *T. almeriensis* *T. dimorphus* *T.obliquus*	No (authorized pursuant to the requirement) -	47% [77] 50% [78] 37% [79]	[79]
*Tetraselmis* *T. chui*	No (authorized pursuant to the requirement) dried: Authorized novel food -	35–40% [80]	[81]

**Table 3:** Categorized overview of literature on microalgae-based food applications published since 2017, including the microalgal species used, the developed product, a general study description, and key findings.

Category	Algae Species	Product	Description	Key Findings	Source
Addition to baked goods	*Arthrospira platensis*, *Chlorella vulgaris*, *Tetraselmis suecica*, *Phaeodactylum tricornutum*	Wheat cookies	Addition of 2%, 6%	+protein +antioxidants Stable color texture: 8 weeks	[82]
	*Haematococcus pluvialis*	Wholemeal (wheat, barley, oat) cookies	Addition of 5%, 10%, 15% astaxanthin powder	+antioxidants −hardness −glycemic response	[83]
	*Chlorella vulgaris*	Wheat flower bread	Addition of 1–5%	0 fermentation 1–3%: +dough rheology 4–5% +aging −dough rheology, −flavor,	[84]
	*Nannochloropsis gaditana*, *Chlamydomonas* sp.	Gluten-free bread	Addition of 1%, 3%	+protein +lipid +ash +Fe +Ca +linolenic acid +ω6/ω3 +color best sensory acceptance: 3% *N. gaditana*	[85]
	*Arthrospira platensis*	Sourdough “Crostini”	Addition of 2%, 6%, 10%	0 fermentation +protein +antioxidants −dough volume −sensory acceptance: 2%	[86]
	*Isochrysis galbana*, *Tetraselmis suecica*, *Scenedesmus almeriensis*, *Nannochloropsis gaditana*	Wheat bread	Addition of 0.4%	+ color 0 texture	[87]
Addition to pasta	*Arthrospira platensis*	Gluten-free pasta	Addition of 1–15%	0 texture +antioxidants 0 cooking quality Best sensory acceptance: 2%	[88]
	*Dunaliella salina*	Pasta	Addition of 1–3%	+protein +fat +ash +minerals +color +cooking quality −carbohydrates −dough strength −sensory	[89]
	*Arthrospira* sp.	Pasta	Addition of 3% in alginate microencapsulated *Arthrospira*	+antioxidants: higher with microencapsulation +consumer acceptance 0 cooking quality	[90]
	*Nannochloropsis* sp.	Dry pasta	Addition of 10%, 20%, 30%, 40% to wheat flour	Pasta structure ok until: 30% +antioxidants +EPA +ω6/ω3	[91]
Addition to other food	*Arthrospira* sp., *Chlorella* sp., *Tetraselmis* sp.	Broccoli soup	Addition of 0.5%, 1%, 1.5%, 2%	+antioxidants Sensory acceptance best: 0.5%	[92]
	*Arthrospira platensis*	Ice cream	Addition of 1% *Arthrospira* microencapsulated in maltodextrin and arabic gum	+protein +antioxidants: higher with microencapsulation +sensory acceptance: less seeweed taste	[93]
	*Arthrospira platensis*	Corn Extrudates	Addition of 2%, 4%, 6%, 8%	0 consumer acceptance +protein +fat +fiber +ash +color +softness −expansion	[94]
	*Arthrospira* spp., *Chlorella vulgaris*	Green smoothie	Addition of 2% to mix of cucumbers, grapes, broccoli	+vitaminB12 *Arthrospira* outperformes chlorella taste +vitaminC degradation	[95]
Substrate for fermentation	*Arthrospira platensis*	Yogurt	Addition of 0.25%, 0.5%, 0.75%, 1%	+fermentation 0 texture 0 sensory acceptance +water holding −whey syneresis +color +antioxidants	[96]
	*Arthrospira platensis*	*Lactobacillus plantarum* fermentation	*A. plantensis* served as a suitable substrate	+antioxidants 0 digestibility	[97]
	*Arthrospira platensis*	Vegan kefir	Addition of 0.25%, 0.5%	+antioxidants +lactobacillus (Lb,)/lactococci −pH	[98]
	*Arthrospira platensis*	Feta-type (BAF) cheese	Addition of 0.1%, 0.5%, 1%	+*L. casei* +protein +beta-carotene 0 texture	[99]
	*Arthrospira platensis*	Vegetal soybean drink	Addition of 9%	+Lactiplantibacillus +protein +antioxidants +digestibility	[100]
Meat alternative	*Auxenochlorella protothecoides*	Extruded meat analogs	Microalgal biomass (14%, 29%, 44%) + Soy protein concentrate (SP): fibrillary textured extrudates with High-Moisture Extrusion Cooking	+vitamins +fat +tenderness best result: 30% Microalgae −texture	[101]
Addition to meat	*Arthrospira* sp., *Chlorella* spp.	Fresh pork sausages	Addition of 1% instead of soy protein	+color +aminoacids +Water holding capacity	[102]
	*Arthrospira* sp., *Chlorella* spp.	Fermented Spanish „Chorizo” sausages	Addition of 3% instead of soy protein	+aminoacids +color +hardness +adhesiveness +gumminess	[103]
Addition to meat alternatives	*Arthrospira* sp., *Chlorella* sp. (yellow)	Plant-based meat patties	Addition of *Arthrospira* (0.5%, 0.7%, 1%) and Chlorella (1%, 2%, 3%) to plant-based-patties (TVP + IPP + IWP)	+color +ash +texture +minerals +antioxidants 0 sensory 0 protein	[104]
Structuring ingredient	*Arthrospira platensis*	Soy protein isolate hydrogel (SPI)	Addition of lysated *Arthrospira*: 1–7%	+rheological properties +mechanical properties	[105]
Texture ingredient	*Arthrospira platensis*	Ice cream	Addition of *Arthrospira* extract containing phycocyanin	+emulsifying & stabilizing activity 0 costumer acceptance	[106]

Legend: + Increased − Decreased, 0 Unchanged, TVP: Textured Vegetable Protein, IPP: Isolated Protein, IWP: Isolated Wheat Protein, ω6/ω3: Omega-6 to Omega-3 Ratio.

**Table 4:** Studies on technofunctional properties of algal proteins.

Strain	Techno-Functionalty	Study	Findings	Ref.
*Chlorella protothecoides*	Emulsification properties	Emulsifyig properties of untreated and hydrolyzed insoluble microalgal protein fractions were studied.	Flocculation-induced creaming suggests suitability for concentrated emulsions; modification (e.g., thermal-acid hydrolysis) enhances functionality.	[121]
*Arthrospira* sp.	Foaming Capacity and Stability	The foaming capacity and stability of protein extracts were evaluated at different pH levels and compared in triplicate to flaxseed and whey protein isolates.	Microalgal protein extracts exhibit good foaming capacity and stability, maintaining performance across pH levels and surpassing flaxseed in stability.	[122]
*Arthrospira platensis*	Gelling properties	The gelling properties of Arthrospira pl. protein isolate were studied using dynamic viscoelastic measurements.	Algal proteins form gels with tunable textures, influenced by heat, pH, and enzymatic modifications. A temperature-dependent gelation process above ~60 °C leads to irreversible network formation through hydrophobic, electrostatic, and hydrogen-bonding interactions.	[123]
*Arthrospira* sp.	Interfacial stabilization	Protein adsorption at oil–water interfaces were investigated Tested different pH, salt concentration, protein concentration and oil polarity	Microalgae proteins reduced interfacial tension better than several animal proteins, Strong, elastic interfacial layers formed near the isoelectric point, faster adsorption at medium pH and higher ionic strength	[124]
*Chlorella vulgaris* *Arthrospira platensis*	Solubility	Covalent conjugation of algal proteins with polysaccharides through the Maillard reaction and non-covalent complexation via complex coacervation have been studied to enhance the techno-functional properties of algal proteins, particularly improving their solubility.	The Maillard reaction can significantly enhance the solubility of algal proteins by attaching hydrophilic carbohydrate groups, improving their water affinity and altering their electrostatic charge. This increased solubility in protein–polysaccharide conjugates, such as *Arthrospira* protein concentrate–maltodextrin, can make algal proteins more versatile for food applications.	[125]
*Tetraselmis suecica*	Surface Activity	The fractions’ impact on surface tension was assessed using an automated drop tensiometer, which applies the Young–Laplace theory to measure interfacial tension.	Algal fractions showed comparable or superior functionality to whey protein isolates, with retentates displaying enhanced surface activity at air–water and oil–water interfaces. This is attributed to hydrophobic compounds and molecular complexes acting like Pickering particles.	[126]
*Arthrospira platensis*	Water and Oil Adsorption Capacity	The water and oil absorption capacity of algal protein isolate was measured using centrifugation-based methods, with results expressed as grams of absorbed water or oil per 100 g of sample.	Algal protein isolate demonstrated good water absorption capacity, with the highest value at pH 10, lower than that of commercial soy protein isolates. It also exhibited a high oil absorption capacity, surpassing soy protein isolates, making it valuable for enhancing texture and flavor retention in food applications.	[127]

**Table 5:** Examples for utilization of various reactor types and published results with respect to final dry cell weight (DCW) for food-grade microalgae.

Cultivation Method	Reactor Type	Strain Name	Cultivation Conditions	Maximal DCW [g/L]	Ref.
Open System	Raceway reactor	*Chlorella variabilis*	15 days, outdoor batch cultivation	1.1 g/L	[141]
	Raceway pond	*Arthrospira platensis*	Semi-continuous cultivation in the 8000 L raceway pond inside greenhouse	0.6–0.9 g/L	[142]
	Pond Reactor	*Arthrospira platensis*	12 days, fed batch cultivation	1.5 g/L	[143]
Closed System	Tubular PBR	*Arthrospira platensis*	8 days fed batch cultivation	3.5 g/L	[143]
	Flat panel gas-lift bioreactor	*Chlorella sorokiniana*	7 days sterile cultivation with LED illumination	1.3 g/L	[144]
	External illuminated PBR	*Chlorella* sp.	20 days batch cultivation in a single Photobioreactor	5 g/L	[145]
	70 L flat-panel vertical photobioreactor	*Chlorella sorokiniana*	13 days semi-continuous cultivation	2.8 g/L	[146]
	2 L Stirred PBR	*Chlorella vulgaris*	14 days cultivaton on low nitrogen media	0.5 g/L	[147]
	34 L loop photobioreactor illuminated by sunlight	*Desmodesmus* sp.	12 days cultivated an aerated with 10% CO_2_,	1.9 g/L	[148]
	2.5 L Stirred PBR	*Euglena gracilis*	14 days photoautotrophic cultivation	5 g/L	[149]
	2.5 L Stirred PBR	*Euglena gracilis*	Heterotrophic cultivation at 27 °C after 7 days of cultivation	10 g/L	[149]
	External illuminated PBR	*Chlorella vulgaris ESP-31*	Photo-heterotropic cultivation on modified Bristol medium over 4–5 days	4 g/L	[145]
	External illuminated PBR	*Chlorella vulgaris ESP-31*	Mixotrophic cultivation on modified Bristol medium over 4–5 days	3 g/L	[145]
	External illuminated PBR	*Chlorella vulgaris ESP-31*	Phototrophic cultivation on modified Bristol medium over 4–5 days	2 g/L	[145]
	External illuminated PBR	*Chlorella vulgaris ESP-31*	Phototrophic cultivation on Basal medium over 4–5 days	5 g/L	[145]
	Vertical Flat Plate PBR	*Odontella aurita*	Stationary phase biomass concentration under increase of nitrogen and phosphor concentrations of the medium after 10 days and under low light illumination	6.8 g/L	[150]

**Table 6:** Comparison of various techniques for microalgal harvesting, adopted from Alam et al. [151]. The economic characteristics presented here are based on a semi-quantitative assessment of reported energy demand, material consumption, and downstream processing intensity in the literature.

Method	Principle	Advantages	Disadvantages	Process Time	Cost	Energy Requirement	Recovery Efficiency	Ref.
Flocculation	Destruction of negative charges on the surface of microalgal cells and change in dispersion stability in the solution	Avoid pollution Efficient harvesting Diversity of flocculation methods	Flocculation capacity is affected by many factors (such as microalgal species, pH value, etc.) Flocculation-agents might be problematic for food/feed applications	Short—Medium	Low—High	Low—High	High	[163,164,165,166,167,168]
Sedimentation	Gravity	Handles large amounts of material No scale-up issues	Flocculation as a necessary pre-treatment Long time	Long	Low	Low	Low	[169,170,171,172]
Flotation	Reverse sedimentation, by collecting rising bubbles or low-density solid particles	High overflow rate Low run time Small footprint Higher concentration factor	High area-to-volume ratio Relies on the flow rate of air	Short	Medium	Low	Medium	[173,174,175,176]
Centrifugation	Centrifugal force	Not species-specific Simple operation Operates in batch or continuous mode Relatively efficient in processing large quantities of microalgae	Effects of gravity and shear forces	Short	High	High	Medium—High	[177,178,179]
Filtration	Cell concentration by using membrane methods	No damage to cells No added chemicals Low shear force Easy to use	Species dependence Cost (ultrafiltration and microfiltration are expensive) Membrane fouling	Short	High	Low	Low—High	[180,181,182]

**Table 7:** Cell disruption techniques for microalgae, including their underlying principles, advantages, limitations, and energy requirements.

	Method	Principle	Advantages	Disadvantages	Energy Requirement	Species Dependent Efficiency	Ref.
Mechanical (physical)	Bead Milling	Collision between high-speed rotating steel, zirconium, glass, or ceramic beads with microalgal cells to cause mechanical cell damage.	Low energy input Efficient disruption Easy scale-up High productivity	Low selectivity Moderate energy consumption Severe conditions Temperature increase Formation of particulates	Medium	*Arthrospira* (moderate), *Chlorella* (high), *Dunaliella* (high) *Haematococcus* (high)	[185,186,187,188,189]
	Pulsed electric field (PEF)	Transient membrane permeabilization through cell electroporation, electrophoretic movement of charged species into the cell, disruption of the lipid bilayer structure of the cell membrane, consequently allowing the diffusion of molecules of a certain size (e.g., small molecular weight proteins) out of the cell.	Proteins with greater stability Easy scale-up Soft conditions High selectivity No particulate formation Eco-friendly Avoids toxic solvents	Moderate energy consumption Medium should be free of ions Medium cannot be conductor Unable to recover chloroplast proteins High energy input	Medium	*Arthrospira* (moderate), *Chlorella* (high), *Dunaliella* (high), *Haematococcus* (moderate)	[190,191,192,193,194]
	High-pressure homogenization (HPH)	Utilization of high pressure (about 200–1200 bar) to promote turbulence, liquid shear stress, and friction, leading to rapid cell rupture, also possible with highly resistant cell wall structure.	Highly effective method High extraction yields Efficient disruption Easy scale-up	High cost Low selectivity High energy consumption Severe conditions Temperature increase Formation of particulates	High	*Arthrospira* (high), *Chlorella* (high), *Dunaliella* (high), *Haematococcus* (high)	[185,194,195,196,197]
	Ultrasonication	high-frequency sound waves (up to 15–20 kHz) induce ultrasonic-based shear forces which induces bubble cavitation over repeated cycles, the cavitation bubbles expand to a critical size before collapsing, resulting in the release of substantial energy. Process can lead to disruption of microalgal cell walls and membranes.	Simple High cell disruption Less need of downstream processing Ensures purity of final product	High power consumption Low disruption efficiency Low selectivity Severe conditions Temperature increase Difficult scalability	Medium	*Arthrospira* (high), *Chlorella* (moderate), *Dunaliella* (high), *Haematococcus* (low)	[187,198,199,200,201,202,203]
	Microwave	Electromagnetic fields cause rapid heating of intracellular water, generating pressure that can disrupt or destroy microalgal cell walls.	Less solvent consumption Reduced operational cost Enhanced extraction rate Short processing time Acceptance of higher cell concentration Easy scale-up Lower power consumption Efficient disruption	Moderate energy consumption Low selectivity Protein denaturation	Medium	*Arthrospira* (high), *Chlorella* (high), *Dunaliella* (high), *Haematococcus* (low)	[204,205,206,207,208]
Non-Mechanical (chemical/biological)	Chemicals	Solvents, acids, bases, salts and other chemicals can interact with components of the microalgal cell wall, causing the cells to deform and prompting them to rupture.	Low energy input High selectivity Moderate conditions	Slow process Not eco-friendly Contamination by reagents Protein degradation Limited protein recovery	Low	*Arthrospira* (high), *Chlorella* (high), *Dunaliella* (high) *Haematococcus* (high)	[209,210,211,212,213]
	Enzymatic treatment	Hydrolytic Enzymes addition facilitates cell wall lysis and rupture.	Eco-friendly Low energy consumption Non-hazardous Soft conditions	Slow process Difficult scale-up High Costs Product inhibition Proteolytic protein destruction limited disruption efficiency complex enzyme mixtures required.	Low	*Arthrospira* (moderate), *Chlorella* (high), *Haematococcus* (high)	[195,211,214,215,216,217,218,219]

**Table 8:** General methods for protein enrichment applied in microalgal protein extraction.

Principle	Example	Results/Yield	Ref.
Ammonium sulfate precipitation	Precipitation of C-phycocyanin from *Arthrospira* extract, using ammonium sulfate as precipitating salt.	Recovery (83.8%) was calculated from total C-phycocyanin content before and after purification based on spectrophotometric concentration measurements. Purity (0.88) was determined as the absorbance ratio A620/A280.	[223]
Aqueous multiphase systems	Concentration of a cell lysate of *C. pyrenoidosa* in a three-phase partitioning system	Protein extract yield of 78% *w*/*w* in middle phase was obtained	[224]
	aqueous two-phase system (ATPS) based on 1-butyl-3-methylimidazolium bromide—dipotassium hydrogen phosphate for the separation of *Parachlorella kessleri* proteins	Proteins mainly partitioned into the ionic liquid-rich top phase, while carbohydrates accumulated in the salt-rich bottom phase	[225]
	ATPS consisting of polyethylene glycol (PEG)/potassium phosphate were used to concentrate phycacyanine from *Arthrospira platensis*	100% recovery and a purification factors of 2.1 was achieved	[226]
Ethanol (EtOH) treatment	Generation of a decolourized protein meal of *Chlorella pyrenoidosa* by high pressure homogenization using Ethanol as solvent.	Effective chlorophyl removal and improvement in protein related functionality such as foaming, emulsification and gelation	[227]
EtOH/acetone treatment	Disrupted cells of *Chlorella protothecoides*, with a protein content of 48%, were treated with EtOH/acetone to generate colorless protein concentrates from both soluble and insoluble protein fractions.	Colorless protein concentrates were obtained with a protein content of 46% for the soluble fraction and 67% of insoluble fraction.	[228]
PH precipitation	*Arthrospira platensis* cell lysate was precipitated at pH 3.5 and diafiltered	11.7% of initial protein content in cells were recovered	[229]
	*Arthrospira platensis* was disrupted via sonication and precipitated by pH shift to 3.89.	75.2% protein were recovered with a purity of 80%.	[221]
	Chlorella sp. Soluble protein was precipitated at pH 3.5	Soluble fractions showed increased protein contents of 57–67%	[230]
	Solid-free alkalic slurry of disrupted *Chlorella sorokiniana* was precipitated with sulfuric acid to the isoelectric point	98.7% were recovered from the protein quantified in the lysate with a purity of 41.4%	[231]
	*Chlorella vulgaris* was disrupted at pH 7 and 12 with a high-pressure homogenizer with subsequent pH-precipitation	Protein solubilization at pH 7 and 12 with 71% and 98% recovery yields, respectively. After acidic pH-shift, 76% were recovered for pH 12 and 57% for pH 7. Dried protein powders could only be solubilized under basic conditions	[220]
Ultrafiltration	*Chlorella vulgaris* was disrupted at pH 7 and 12 with a high-pressure homogenizer and soluble proteins were recovered by tangential ultrafiltration in 300 kiloDalton (kDa) cut-off	The major fraction of proteins remained in retentate (87% and 95% for pH 7 and 12, respectively	[220]
	Proteins in Cell free *Arthrospira* extract were concentrated by using ultrafiltration with a 3 kDa cut-off	92% of total proteins were recovered in the retentate	[232]
	extraction and isolation of phycocyanin with membrane-based microfiltration and subsequent ultrafiltration	Microfiltration yielded >60% phycocyanin recovery, and ultrafiltration resulted in >99% phycocyanin retention and a 1.5-fold increase in purity.	[233]
	Fractionation of peptide from Microalgal Protein Hydrolysate (*Nannochloropsis gaditana*) using a Two-Stage Cross-Flow Ultrafiltration Membrane (10/5 kDa)	Peptide transmission of 79.13% was achieved in the permeate through the applied membrane system	[234]

**Table 9:** Typical treatments used in protein modification and processing.

Treatment	Method	Energy Requirements	Results	References
Chemical	Succinylation	Low	Increased soluble protein fraction in soybean	[254]
			Significant higher solubility in *Arthrospira platensis* protein isolate	[255]
			Higher solubility, increased emulsifying properties in oat protein isolates	[256]
	Glycosylation	Low	Improved emulsion thermal stability of peanut protein isolate	[241]
	Acetylation	Low	Increase in water and oil absorption capacity, foam stability, and digestibility for mung bean protein isolates	[242]
	Covalent linkage with tannic acid	Low	Higher antioxidant activity of *Arthrospira* protein	[257]
Heat	Ohmic heating	Medium	tailored denaturation and aggregation of whey protein isolates	[243]
	Microwave treatment	Medium	increase in gel hardness and water-holding capacity for protein-polysaccharide gels	[258]
Enzymatic	Transglutaminase	Low	PH-dependant increase in emulsion and foaming capacity of wheat gluten emulsion	[259]
	Proteases	Low	Higher solubility, emulsifying, and foaming capacities of pea protein	[247]
			Significant increase in solubility and functionalities of rice dreg protein	[260]
	Protein-glutaminase	Low	Increase in solubility and emulsification, and decrease in allergenicity of wheat gluten	[248]
Physical/Mechanical	High-pressure processing	High	Improve in solubility and gelation for plum seed, wolfberry jujube seed, and hemp seed protein isolates	[250]
	High-pressure processing combined with glycosylation	High	Phycocyanin reached better surface activity, solubility, and color stability.	[261]
	Ball milling	Medium	Increase in gel strength and water holding capacity of soybean protein isolate	[251]
	Pulsed electric field	Medium	Increased solubility and hydrophobicity of soybean protein isolates	[262]
	ultrasound	Medium	Increased foaming properties of *Arthrospira* proteins	[263]
	High Moisture Extrusion Cooking	High	*Auxenochlorella* protein performs poorly in high-moisture extrusion compared to soy or pea protein	[253]

**Table 10:** Policy priorities to accelerate microalgal proteins as sustainable food ingredients, along with expected effects.

Priority	Policy Action	Responsible Actors	Intended Impact
1	Establish long-term, coherent regulatory frameworks for microalgae-derived food ingredients	EU institutions; national authorities	Reduce regulatory uncertainty and de-risk industrial investment in microalgal protein technologies
2	Define harmonized sustainability, safety, and quality criteria specific to microalgae-based foods	EU regulators; standardization bodies	Enable consistent life-cycle assessment, facilitate Novel Food approval, and support evidence-based policy decisions
3	Standardize analytical methodologies for protein quantification, digestibility, and functional performance	Research agencies; regulatory bodies	Improve comparability across studies and strengthen regulatory and consumer confidence
4	Support pilot- and demonstration-scale infrastructure for food-grade microalgae cultivation and protein processing (TRL 6–8)	Public–private partnerships	Validate scalability and accelerate translation from laboratory to industrial production
5	Implement long-term, stage-gated public funding schemes for microalgal protein research	National and EU funding agencies	Enable sustained strain development, cultivation optimization, and downstream innovation
6	Incentivize knowledge transfer and scale-up from academia to start-ups and SMEs	Public innovation agencies; private investors	Accelerate translation from TRL 3–6 and strengthen a competitive European microalgal ecosystem
7	Mobilize patient capital and blended-finance instruments for capital-intensive scale-up phases	Public banks; private equity	Support industrial deployment and reduce financial risk in early commercial stages
8	Introduce fiscal incentives and risk-sharing mechanisms for sustainable microalgal production infrastructure	Governments	Lower capital expenditure barriers and stimulate private-sector participation
9	Promote equitable technology transfer and capacity building between Global North and Global South	Governments; international organizations	Support global food security and inclusive bioeconomy development

## Data Availability

No new data were created or analyzed in this study. Data sharing is not applicable to this article.
